# Are humans facing a sleep epidemic or enlightenment? Large-scale, industrial societies exhibit long, efficient sleep yet weak circadian function

**DOI:** 10.1098/rspb.2024.2319

**Published:** 2025-02-26

**Authors:** David Ryan Samson, Leela McKinnon

**Affiliations:** ^1^ Department of Anthropology, University of Toronto Mississauga, Mississauga, Ontario L5L 1C6, Canada

**Keywords:** sleep, chronohygiene, circadian function, mismatch, evolutionary medicine

## Abstract

The Centers for Disease Control and Prevention declared sleep-related problems to be a public health epidemic. With the advent of biometric sleep tracking technology taking the sleep lab into the field, the study of human sleep is now global, and these new datasets show contrasting findings. Previous reports suggest sleep in small-scale, non-industrial societies to be short and fragmented yet characterized by greater circadian rhythmicity. However, the role of circadian rhythm indicators in understanding global variations in human sleep patterns remains unclear. We examine population-level sleep studies (*n* = 54) using polysomnography and actigraphy to test the sleep restriction epidemic hypothesis, which posits that labour demands and technological disruption in large-scale, industrial societies have reduced sleep duration. We used an actigraphy-generated circadian function index from both non-industrial and industrial societies (*n* = 866) to test the circadian mismatch hypothesis, which suggests that poor chronohygiene in regulated environments misaligns circadian rhythms in industrial societies. In rejection of the sleep restriction epidemic hypothesis, our results show that industrial societies experience the longest, most efficient sleep, whereas in support of the circadian mismatch hypothesis, sleepers in non-industrial societies are characterized by the greatest circadian function.

## 1. Introduction

Sleep restriction, increasingly described as an epidemic in the Global North— covering Europe, North America, and developed parts of Asia [1,2]—impacts an estimated 92 million individuals in the United States alone [3], with the economic cost in some nations nearing 1% of gross domestic product (GDP) [4,5]. A study across eight African and Asian countries shows about 17% of adults suffer from severe sleep problems, suggesting sleep restriction is a global issue [6]. This perspective, termed the ‘sleep restriction epidemic hypothesis’, has sparked heightened concerns among the general populace [3]. However, evidence from small-scale, non-industrial societies, often without electricity, challenges this ‘sleep restriction epidemic hypothesis’ and sparks debate over sleep characterization and measurement across societies [7,8]. Technological disruptions such as electronic devices and work demands are seen as major contributors to sleep deprivation, but cross-societal research indicates a need for broader investigation into sleep patterns [9,10].

The development of actigraphy, a non-invasive method measuring physical activity levels via a wristwatch-like device, has significantly advanced sleep research [11]. This technology, validated against polysomnography (PSG), has enabled reliable sleep measurement in humans without access to sleep labs, particularly in dynamic field environments. This breakthrough has paved the way for previously unexplored avenues of cross-cultural sleep research [11]. Alternative approaches to quantifying sleep patterns, such as longitudinal time-use studies in the United Kingdom, have demonstrated that contemporary sleepers are obtaining an additional 43 min of sleep compared with the 1970s, attributed to earlier bedtimes. Interestingly, this trend applies across all ages, employment statuses and genders in industrial countries, which are presumed to be most afflicted by the sleep restriction epidemic [12].

This begs the question: has human sleep remained constant throughout history? Human sleep patterns are complex. Various factors influence them, suggesting that sleep duration is not a static, species-wide trait but rather a dynamic variable shaped by environmental, biological and social factors [13,14]. Evidence indicates that both environmental conditions and lifestyle changes—such as access to electricity and increased urbanization—impact sleep [14,15]. For example, rural populations in Brazil, even those with access to electricity, display significant variation in diurnal preference and earlier sleep–wake timing compared with urban populations, challenging the notion that industrialization alone shortens sleep duration [16]. Additionally, small-scale agricultural societies with access to electrification, such as those studied by Ruiz *et al*. [15], exhibit circadian rhythms and sleep durations that closely resemble those of non-electrified populations. By studying diverse subsistence patterns and socioecological settings, we can gain a more refined understanding of human sleep norms that better informs comparative and evolutionary analyses across primate taxa. This broader context underscores the importance of evaluating variability in sleep duration, efficiency and circadian alignment across societies as a critical lens for understanding the evolutionary forces shaping human sleep.

Intriguingly, a clearer pattern emerges from the study of sleep in small-scale, non-industrial societies, particularly among hunter–gatherers and nomadic herders. Initial reports on sleep among equatorial hunter–gatherers reveal surprisingly short sleep durations among the San (6.66 h) [17], Hadza (6.22 h) [18] and BaYaka (5.94 h) [19–21]. Even more striking are findings from the first investigation of sleep among nomadic agropastoral groups; the Himba of Namibia sleep just 5.47 h per night [22]. Consequently, a robust discourse on global human sleep trends, encompassing both sleep quantity and quality, has ensued. Clinicians working with populations in industrialized areas often point to technology as a major factor in sleep restriction. Meanwhile, anthropologists studying non-industrial and particularly forager societies, where such technology is absent, note observations of short and inefficient sleep [8]. Are humans truly sleeping 1−2 h less per night in many countries compared with their ancestors of 50−100 years ago, as some researchers claim [1]? Or is it possible that individuals in the industrial societies experience longer and more efficient sleep than their forebears?

It is essential to differentiate between circadian and sleep disorders [23]. Sleep and circadian rhythm pathologies can be categorized as dyssomnias (difficulty falling or staying asleep) [24] and parasomnias (abnormal sleep-related activities) [25], indicating distinct mechanistic pathways. These disorders are interconnected in a two-way, yet separately identifiable relationship that impacts sleep quality, timing and duration, ultimately leading to daytime difficulties and a decline in overall health and well-being. While ‘optimal’ sleep is characterized by architecture (non-rapid eye movement (NREM) and rapid eye movement (REM)) and assessed in terms of duration and quality (fragmentation/efficiency) [26], circadian alignment involves synchronizing various organ clocks to the 24 h cycle while maintaining normal phase relationships among them. Misalignment occurs when clocks are synchronized to 24 h but adopt abnormal phase relationships with each other, resulting from environmental (e.g. light and temperature) or social (school, work and leisure activities) factors. This misalignment can disrupt circadian function, influencing daily rhythms of sleep–wake activity, temperature and hormone secretion, potentially compromising health and well-being [27]. These distinctions have been substantiated through experimental evidence. Circadian misalignment is broadly linked to conditions like cancer and depressive disorders [28,29]. It impairs autonomic function, increasing cardiovascular risks [29]. It is associated with increased insulin sensitivity and inflammation, effects observed independently of an individual’s total sleep duration. This distinction is crucial in understanding the unique consequences of misalignment, as demonstrated in studies where circadian misalignment was shown to exacerbate health risks such as diabetes and inflammation, beyond the effects of sleep loss alone [30]. Conversely, short or fragmented sleep is associated with various adverse health outcomes, including hypertension, obesity, kidney disease and infertility [31,32]. Evolutionary mismatch, denoted as ‘mismatch’, refers to a scenario where evolved adaptations no longer provide advantages owing to incongruence with novel environments [33]. For instance, continuous indoor dwelling suppresses potent circadian entrainment cues like light and temperature [34], potentially prompting circadian misalignment. From an evolutionary standpoint, transitioning to an environment that prioritizes secure sleep at the expense of circadian function could lead to a profound state of evolutionary mismatch.

Extensive research has underscored the critical role of circadian rhythms in determining overall health outcomes, with numerous interventions designed to mitigate the adverse effects of circadian misalignment [35–37]. Building on this foundational knowledge, our study seeks to explore the nuanced variations in circadian alignment across diverse populations. By examining these variations, we aim to contribute new insights into how societal and environmental factors influence circadian patterns, potentially informing more tailored and effective public health interventions. A potential solution to reconcile the divergent sleep findings from different societies is a cross-cultural, comparative analysis of sleep and circadian function.

In this study, we present such an analysis to simultaneously evaluate the sleep restriction epidemic hypothesis and the circadian mismatch hypothesis. The circadian mismatch hypothesis posits that non-industrial small-scale societies, living ‘off the grid’, will exhibit robust circadian function. Conversely, populations within industrial agricultural societies will display longer, higher-quality sleep owing to safer and more comfortable resting environments, but at the cost of circadian misalignment stemming from reduced exposure to environmental entrainment factors.

To empirically investigate these hypotheses, we conducted a comprehensive cross-national analysis by integrating reported sleep data from 54 global populations with diverse subsistence strategies, access to electricity and ecological conditions. We assessed sleep duration and efficiency, categorized by society scale (electronic supplementary material, table S1). For instance, among the non-industrial groups analysed (*n* = 10), 30% were foragers, deriving the majority of their calories from hunting and gathering, while 70% were horticulturalists, obtaining most of their sustenance from self-grown sources. Furthermore, 70% lacked access to the electric grid. Concurrently, we performed a comparative examination of circadian function (*n* = 866) using datasets from three society categories: (i) prior research among foragers [18,19], (ii) studies involving non-industrial, small-scale agricultural societies [38,39] and (iii) studies involving large-scale societies in the United States [40]. Detailed population descriptions are provided in electronic supplementary material, table S2.

We model sleep patterns, explicitly testing both the sleep restriction epidemic hypothesis and the circadian mismatch hypothesis. If the sleep restriction epidemic hypothesis holds true, we anticipate lower sleep duration and efficiency among industrial populations. In the second study, we used circadian function index (CFI) to examine the circadian mismatch hypothesis. Should the circadian mismatch hypothesis be supported, we expect heightened circadian function among non-industrial populations.

## 2. Methods

Our analysis encompasses two distinct approaches—previously published sleep quotas derived from both PSG and actigraphy, based on the type of data available and the specific outcomes of interest (see electronic supplementary material, tables S1 and S2). Our models used previously published data. In both studies, posterior distributions for each parameter were summarized using 89% and 95% credible intervals (CI), which provide an intuitive understanding of parameter uncertainty. The Bayesian approach allowed us to directly estimate the probability that industrial societies have sleep and circadian function that differ from non-industrial societies.

### (a) Study 1

Our samples cover a diverse set of cultures from all inhabited continents. We collected meta-data from studies reported between 1967 and 2022, spanning 21 countries: Argentina, Australia, Bolivia, Brazil, Canada, Congo (Democratic Republic), France, Germany, Guatemala, Haiti, Italy, Madagascar, Mozambique, Namibia, The Netherlands, Scotland, Sri Lanka, Switzerland, Tanzania, the United States of America (USA) and Vanuatu (see electronic supplementary material, table S1). This dataset comprises 54 studies, with a total of 5101 participants, and includes both industrial and non-industrial societies. Sleep was measured using PSG and actigraphy, and total sleep duration throughout the night was recorded for each study. In 38 studies, sleep efficiency was also assessed. Sleep duration in non-industrial societies ranged from 5.34 to 7.88 h, while industrial societies reported a range of 6.00−9.17 h.

We selected studies for inclusion in our analysis based on the following criteria: (i) sleep duration and efficiency data were obtained via validated measurement techniques such as PSG or actigraphy; (ii) participants were aged between 18 and 75 years, and those with diagnosed sleep disorders or chronic illnesses were excluded; (iii) only baseline data were included from studies with experimental interventions; and (iv) we excluded studies relying solely on self-reported sleep estimates. By applying these criteria, we aimed to ensure methodological consistency and control for potential confounding factors across populations. We emphasized the integration of studies that clearly reported biological sex and age data. Biological sex at the population level was recorded as the percentage of the study population identified as male. Additional factors considered in the analysis included subsistence strategy, access to electricity, and latitude and longitude of study location.

#### (i) Modelling

Bayesian inference was used to estimate posterior distributions [41]. We used R (version 4.4) [42] and the brms package [43] to make inferences about the shape of the posterior distribution for sleep parameters. Sleep quota values, estimated using a Bayesian Markov chain Monte Carlo (MCMC) approach, generated a *σ—*the equivalent to the predicted standard deviation (s.d.) for all categories combined. The highest density interval (HDI) is the Gaussian approximation for each parameter’s marginal distribution. The plausibility of each value of *µ* after averaging over the plausibility of each value of *σ* is given by the distribution’s mean and s.d. In this case, the percentile interval boundaries correspond to an 89% interval.

Regarding our model’s prior specifications, we reference various sources. Notably, *Principles and Practice of Sleep Medicine*, a key text for sleep clinicians, offers an overview of normal human sleep, suggesting that most young adults report around 7.5 h of sleep on weekday nights and approximately 8.5 h on weekend nights [44]. Furthermore, the National Sleep Foundation’s expert panel consensus recommends 7−9 h of sleep for adults aged 26−64 years for optimal health and well-being [45]. This range effectively captures the variability in sleep needs among the general population, which is why we considered an s.d. of 1 h reflective of the accepted variability around the 8 h mean. Consequently, our sleep model adopted an 8 h sleep duration prior, represented by *µ* = 8 h and *σ* = 1 h. Similarly, sleep efficiency, defined as the time spent actually sleeping while in bed, was guided by the National Sleep Foundation’s optimal health recommendation of 85% [46]. Therefore, our sleep efficiency model’s prior encompassed a *µ* = 85 with a *σ* = 10. While our priors are primarily derived from industrialized populations, they offer a beneficial foundation for initial analysis, with the caveat that broader demographic data are essential for future refinement. Our analytical approach sought to estimate human sleep duration and sleep efficiency, while accounting for pertinent factors such as non-industrial contexts, various methods of sleep quota determination, individual age, biological sex and the country in which the study was conducted, treated as a repeated measure. We used the leave-one-out information criterion (LOOIC) to identify the model with the highest predictive capacity [47]. After model fitting, we found that the method (actigraphy vs PSG) and sample size were not significant drivers and removed them from subsequent models. To address spatial autocorrelation, geographical coordinates were initially included, but did not significantly explain variations in sleep duration or circadian function. Moran’s *I* tests on random intercepts (latitude and longitude) against sleep duration parameters yielded a statistic of −0.0192 (expected under the null hypothesis: −0.0192, *p* = 0.5), indicating no spatial clustering of random effects. Thus, geographical proximity did not influence sleep duration. Including ‘country’ as a random effect sufficiently accounted for geographical variation, reflecting broader socioecological differences beyond spatial proximity.

The most predictive model, according to the information criterion, used *non-industrial* as a factor. The classification into ‘non-industrial’ and ‘industrial’ societies was based on a multifaceted assessment of each society’s technological access, economic structures and lifestyle patterns rather than merely on population size. This assessment included but was not limited to, factors such as electricity access, reliance on technology for daily living, economic dependence on industrial or post-industrial frameworks, and urbanization levels. For example, societies labelled as ‘non-industrial’ typically exhibit minimal reliance on technology, subsistence-based economies and limited access to electricity, reflecting conditions more akin to those experienced by humans for the majority of our evolutionary history. Conversely, ‘industrial’ societies are characterized by high levels of technology use, economic activities centred around manufacturing or services, widespread access to electricity, and urbanized living conditions. This categorization aims to encapsulate the varying environmental and social pressures that may influence sleep patterns. Thus, the terms ‘small-scale’ (non-industrial) and ‘large-scale’ (industrial) are meant to reflect differences in organizational subsistence-related complexity and demographic scale.

Taken together, the model used is as follows:


Sleep Model: Sleep ∼ Society_scale+Age+%male+(1|Country).

The model is designed with hierarchical levels that explicitly account for the hierarchical structure of the data—individuals nested within populations, which in turn are classed as either *society scale* category: non-industrial or industrial. This structure allows us not only to model sleep duration as an outcome of individual and population-level predictors but also to consider the variability within and across these groups. To address potential biases from over- or under-representation of certain population types, our model employs a random effects structure. This approach assigns a unique effect for each population, which is drawn from a common distribution. By doing so, it naturally weights the contribution of each population to the overall analysis, mitigating the influence of populations with disproportionately large or small sample sizes. This method ensures that our global human sleep metric is a balanced representation, not skewed by the data volume from any single group. Furthermore, having *society scale* as a fixed effect in our model directly examines and quantifies the influence of living in different ecological and social environments on sleep duration. This inclusion allows the model to adjust for the differences attributable to the population type, thereby providing a more nuanced understanding of human sleep patterns across varying conditions.

The full dataset, along with all meta-data and more detail of each variable, is available in the Open Science Framework (OSF) data repository: https://osf.io/jy8ch/.

The initial hypothesis and predictions were pre-registered and developed upon for this work in preprint form via: https://www.biorxiv.org/content/10.1101/2020.09.16.299792v1.

### (b) Study 2

Circadian rhythms can be studied in a variety of ways. One external measure of circadian rhythms is through rest–activity patterns measured with wrist-worn actigraphy. In Study 2, we used non-parametric circadian rhythm analysis (NPCRA) [48], which had previously been reported in a variety of actigraphy studies [18,19,38,40,49,50], to calculate an index of circadian consistency, fragmentation and amplitude known as the CFI [46]. The NPCRA technique, which does not assume any pre-defined distribution, generates circadian phase markers. A minimum of six consecutive nights is suggested for NPCRA statistics, and this minimum was reached for each population, providing consistency across all population data presented. CFI incorporates three main parameters: intradaily variability (IV), interdaily stability (IS) and relative amplitude (RA) [46]. Previous clinical work [51] incorporated IS, IV and RA into a single index variable to yield the CFI. IS provides an estimated measure of rhythm stability (ranging between 0 and 1) where 0 is Gaussian noise and where 1 is a perfect rhythm stability from one day to the next. IV is an estimated measure of rhythm fragmentation, with values of 0 indicating a perfectly sinusoidal curve, and 2, Gaussian noise. RA indicates the amplitude of rhythm, where higher values (between 0 and 1) are indicative of a higher amplitude rhythm. IV was inverted and normalized to reflect the degree of rhythm fragmentation, with higher values indicating greater fragmentation. IS quantified rhythm stability over different days, and RA represented the difference between the mean activity levels during the highest and lowest consecutive hours. By merging IV, IS and RA, CFI captures the amplitude and stability of circadian rhythms in a single composite index, ranging between 0 (absence of circadian rhythmicity) and 1 (a robust circadian rhythm). This multidimensional index aims to provide a comprehensive overview of circadian rhythms within individuals' daily contexts, considering both amplitude and stability components.

A total of 866 participants from five different countries were used to derive CFI values (see electronic supplementary material, table S2). CFI is calculated using NPCRA to derive three measures of circadian function: RA, IS and IV. These parameters were then used to derive CFI using previously described techniques [52]. Specifically, IV values were inverted and normalized between 0 and 1, with 0 being a noise signal, and 1 a perfect sinusoid. Finally, CFI was calculated as the average of these three parameters. Consequently, CFI oscillates between 0 (absence of circadian rhythmicity) and 1 (a robust circadian rhythm).

We employed a Bayesian framework to model the CFI across industrial and non-industrial populations. This analysis was conducted using the brms package [43] in R, which implements Hamiltonian Monte Carlo (HMC) sampling via Stan to estimate the posterior distributions of the model parameters. The model was structured to include s*ociety scale* (industrial vs non-industrial), age and sex as fixed effects.

In constructing the model, we incorporated informative priors to guide the estimation process. For the CFI intercept, we used a normal prior informed by the empirical findings of Ortiz-Tudela and colleagues [52], who observed CFI values ranging between 0.43 and 0.73 in a homogeneous population. Based on these observations, we applied a prior with a mean of 0.58 and an s.d. of 0.1, ensuring the model captured the range of CFI values typically observed in previous studies. The model is specified as follows:


CFI Model: CFI ∼ Society_scale+Age+Sex.

## 3. Results

### (a) Sleep duration is longer among industrial societies

**Figure 1.**
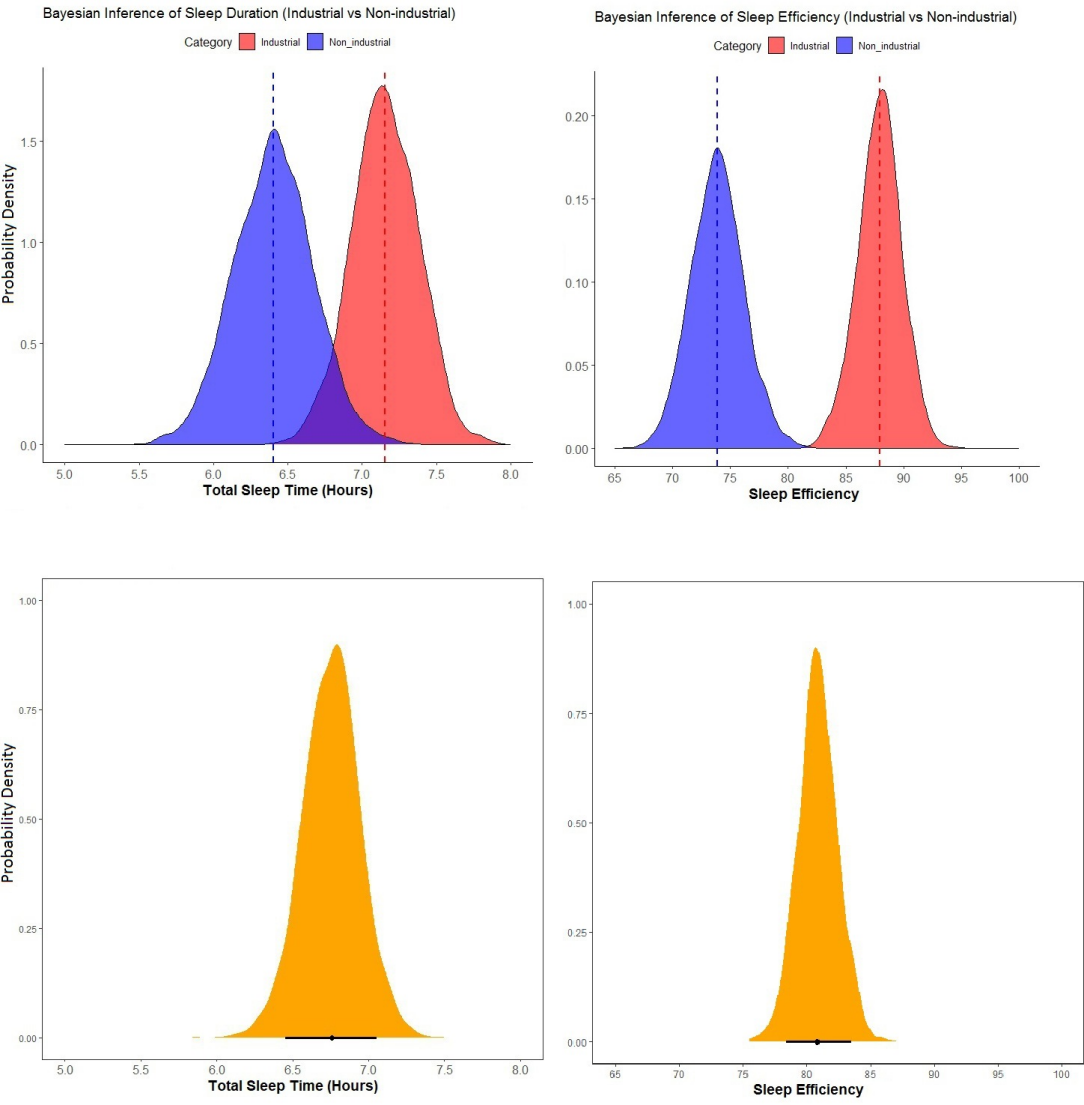
Density plots of Bayesian inference for sleep duration and efficiency using Markov chain Monte Carlo (MCMC) approach. The top left plot shows the probability density for sleep duration in industrial (red) and non-industrial (blue) populations. Industrial populations have longer expected sleep durations compared with non-industrial populations, as reflected in their respective density distributions. The bottom left plot shows the density distribution for the expected human sleep (yellow), representing the combined estimate across all populations in the study. Similar patterns hold for the top right plot, which shows the probability density for sleep efficiency % among societies, and the bottom right plot shows the mean for the all societies. The *y*-axis in all plots represents the probability density, and dashed vertical lines indicate the mean sleep duration and efficiency % for each category.

In Study 1, we aimed to derive a more nuanced understanding of sleep patterns across diverse populations, discerning the influences of multiple variables. Our subsequent results, as presented in figure 1, offer insights into the posterior distribution of human sleep duration. Specifically, across all populations, the posterior distribution of human sleep duration was characterized by a mean (*µ*) of 6.78 h, with an s.d. (*σ*) of 0.18 h, and a credible interval (89% CI) spanning from 6.5 to 7.06 h. Moreover, our results show that non-industrial sleep duration posteriors are *µ* = 6.4 h (*σ* = 0.27, 89% CI = 5.88–6.99) and industrial sleep duration posteriors are *µ* = 7.1 h (*σ* = 0.22, 89% CI = 6.70–7.58). Additionally, we plot and predict sleep parameters among the non-industrial and industrial populations while controlling for age, sex and country. As shown in table 1, and displayed in electronic supplementary material, figure S1, while correcting for age and sex, non-industrial status negatively influences sleep duration.

**Table 1.** Markov chain Monte Carlo (MCMC) posterior estimations with 89% and 95% credible intervals (CI) of sleep duration across global, cross-cultural populations. 'Industrial' is the reference category for society scale. Positive coefficients indicate greater sleep duration, while negative coefficients indicate lesser sleep duration. The model accounts for country as a random effect. Columns for '% negative' represent the proportion of posterior samples in each direction, and also highlight level of strong support (>95%).

predictor	estimate (s.e.)	89% CI	95% CI	% negative (support)
intercept	7.16 (0.22)	(6.80, 7.51)	(6.71, 7.59)	
society scale: non-industrial	−0.75 (0.34)	(−1.31, −0.23)	(−1.44, −0.08)	98.4 (strong)
age	−0.19 (0.07)	(−0.30, −0.09)	(−0.32, −0.06)	99.8 (strong)
sex (% male)	−0.13 (0.06)	(−0.22, −0.03)	(−0.24, −0.01)	98.5 (strong)

To assess potential differences in sleep duration between industrial and non-industrial populations, we examined the posterior distributions of each group after Bayesian model fitting. On average, industrial populations displayed a longer sleep duration than non-industrial populations, with an estimated mean difference of 0.75 h. The group-level random effect for country (0.73 ± 15), 89% CI = 0.49−1.05) indicates substantial variation in sleep duration attributable to country-level differences, highlighting the importance of contextual and cultural factors in shaping sleep patterns across populations. This variability underscores the necessity of accounting for country as a random effect to appropriately model sleep duration and enhance the generalizability of the findings.

We have included both 89% and 95% credible intervals in our analysis. This dual reporting provides a more comprehensive view of the robustness of our findings, allowing readers to assess the results under different thresholds of uncertainty. Also, to further operationalize and clarify the strength of our findings, we adopted a systematic approach to evaluate support for regression coefficients. Specifically, we assessed the proportion of posterior samples in which coefficients were negative. We categorized support as weak (85−90%), support (90−95%) or strong (>95%). These thresholds were applied to the proportion of samples in the predicted direction, providing an intuitive measure of the reliability of the estimated effects.

### (b) Sleep efficiency is greater among industrial societies

**Figure 2.**
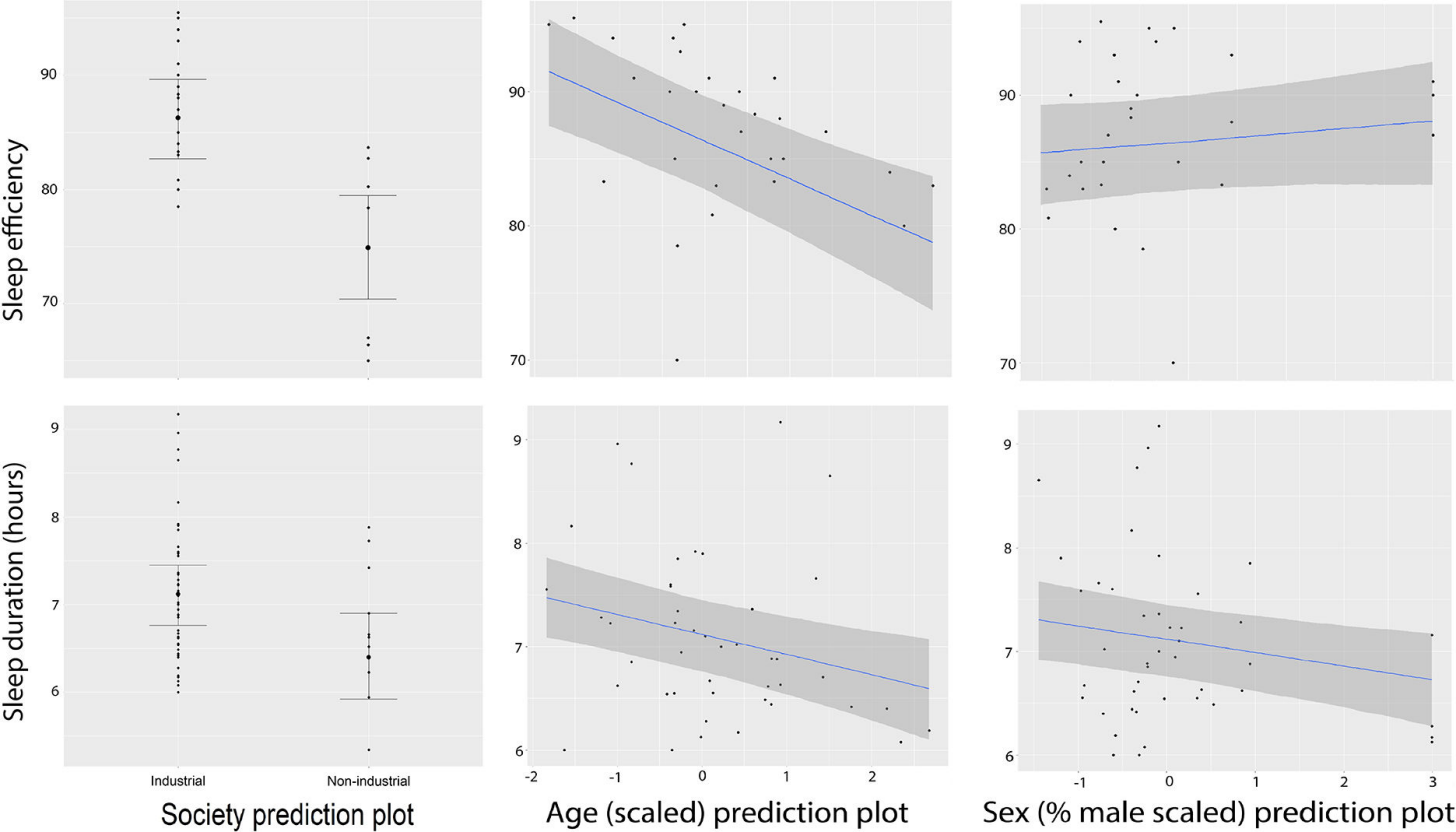
Sleep duration and efficiency model plots using Markov chain Monte Carlo (MCMC) approach. The left panels showcase actual sleep data (see electronic supplementary material, table S1), categorizing observations into non-industrial and industrial populations for both sleep efficiency % (top) and sleep duration (bottom). These plots represent observed data, with error bars indicating standard deviations. In the predictive plots (middle and right panels), darker-shaded areas represent 89% credible intervals, elucidating the uncertainty in our predictions related to age and sex influences on sleep efficiency and duration. The statistical significance and relationships between sleep metrics and various predictors (e.g. societal scale, age, sex) are inferred from posterior distributions.

Likewise, our investigation into sleep efficiency—a measure denoting the proportion of time an individual spends sleeping while in bed—yielded valuable insights. The posterior distribution of human sleep efficiency demonstrated a mean of *µ* = 80.9, an s.d. of *σ* = 1.56, and a credible interval spanning from 78.5 to 83.5. Notably, non-industrial and industrial populations exhibited sleep efficiency posteriors of *µ* = 73.9 (*σ* = 2.30, 89% CI = 70.3–77.7) and *µ* = 87.9 (*σ* = 1.92, 89% CI = 84.8–90.9), respectively. Additionally, with sleep efficiency as the response variable, we used the same model fit (table 2) to demonstrate that non-industrial status is negatively associated with sleep efficiency (electronic supplementary material, figure S2). Thus, non-industrial status appears to be linked to both sleep duration and efficiency (figure 2). Age negatively influences both sleep duration and efficiency (figure 2). A higher percentage of the study population identified as biologically male negatively influences sleep duration but does not influence sleep efficiency (figure 2). The multilevel model included a random intercept for country to account for potential variability in sleep efficiency across different populations. The estimated s.d. of the country random effect was 5.42 ± 1.46 (89% CI = 3.38–7.89), indicating substantial variation in sleep efficiency attributable to country-level differences. Accounting for this random effect enhances the model’s ability to generalize findings and underscores the necessity of considering country-specific influences when examining sleep patterns.

**Table 2.** Markov chain Monte Carlo (MCMC) posterior estimations and credible intervals (89% and 95%) for sleep efficiency among global, cross-cultural populations. 'Industrial' is the reference category for society scale. Positive coefficients indicate greater sleep efficiency, while negative coefficients indicate lesser sleep efficiency. The model includes a multilevel structure, with country of the study population modelled as a random intercept to account for group-level variability. Column for '% negative' represents the proportion of posterior samples in each direction, and also highlights level of support with strong support designated at >95%.

predictor	estimate (s.e.)	89% CI	95% CI	% negative (support)
intercept	87.91 (1.92)	(84.76, 90.95)	(83.98, 91.57)	
society scale: non-industrial	−14.01 (2.86)	(−18.49, −9.28)	(−19.41, −8.36)	100 (strong)
age	−2.80 (0.77)	(−4.02, −1.55)	(−4.32, −1.22)	99.9 (strong)
sex (% male)	0.64 (0.63)	(−0.36, 1.65)	(−0.58, 1.93)	14.6

Contrasting non-industrial versus industrial populations, the results support differences in sleep parameters. The mean difference of 14% emphasized the significantly higher sleep efficiency in industrial populations. In sum, these results support the idea that there are differences in sleep efficiency patterns across varying population categories. Non-industrial populations tend to manifest lower sleep durations, while larger-scale populations exhibit sleep durations that exceed prediction.

### (c) Circadian function is greater among non-industrial societies

**Figure 3.**
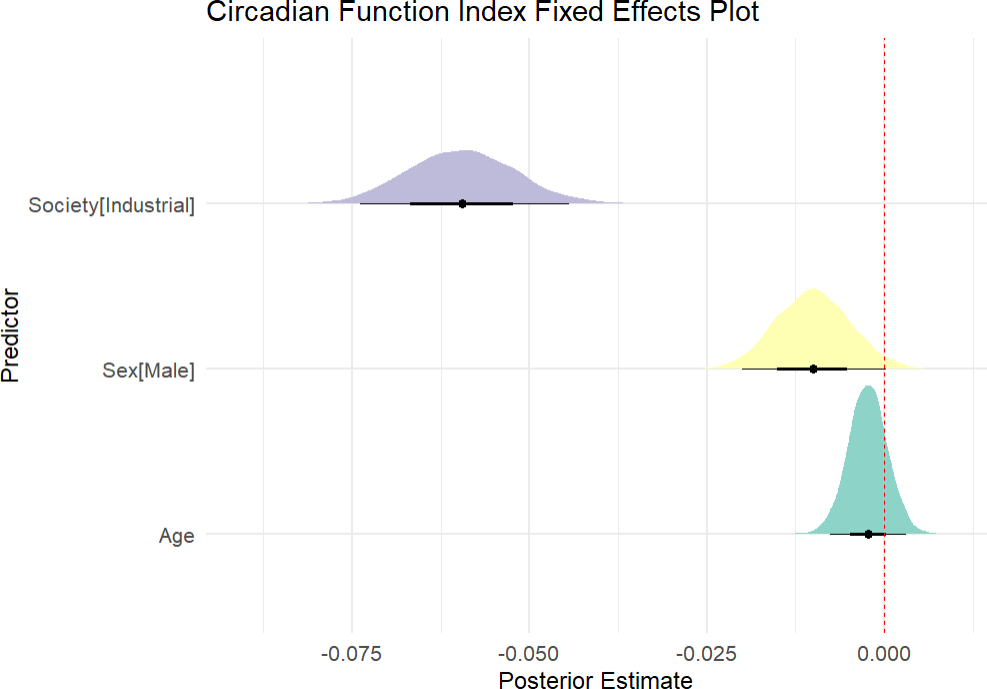
Circadian function index (CFI) model estimates and prediction plots using Markov chain Monte Carlo (MCMC) approach. Non-industrial societies are associated with higher CFI values compared with industrial societies, with the credible interval not overlapping zero. Age has no significant effect on CFI, as its credible interval includes zero, while the effect of sex (male) shows a slight negative association, though its credible interval approaches zero.

Descriptively, the CFI across societal types indicates discernible disparities. Non-industrial populations have a mean CFI of 0.70 (s.d. = 0.01), with values spanning from 0.44 to 0.93 (*n* = 93). In contrast, industrial populations present a lower mean CFI of 0.63 (s.d. = 0.07), with a range of 0.36–0.86 (*n* = 773). These data points reflect a generally higher CFI in non-industrial societies relative to industrial ones. The posterior estimate for society scale (comparing industrial with non-industrial societies) is −0.06 (s.e. = 0.01), with the 89% CI (−0.07, −0.05) and 95% CI (−0.08, −0.04) not crossing zero, suggesting a significant negative association between industrial society and a reduction in circadian function (figure 3). Specifically, 100% of the posterior samples were negative in direction, providing strong evidence for the reduced CFI in industrial societies relative to non-industrial societies. In contrast, age was not a strong driver of CFI, as its posterior estimate was close to zero, with a corresponding credible interval that included zero. The effect of male biological sex showed a slight negative association with CFI, but the credible interval for this effect approached zero, suggesting that, while a weak association exists, it is not strong enough to be conclusive.

## 4. Discussion

Our research revealed a startling juxtaposition between sleep and circadian function across small-scale non-industrial and large-scale industrial societies. People dwelling within non-industrial societies—many of which are local subsistence, non-electric, ‘off-the-grid’ and residing in the Global South— experience shorter, less efficient sleep yet have a greater circadian function. The inverse is also true, where people dwelling within industrial societies—many of which are characterized as heavily reliant on provisioned food, access to electricity, and residing in the Global North—are experiencing longer and more efficient sleep yet reduced circadian function.

On the one hand, our work rejects the *sleep restriction epidemic hypothesis*—the idea that sleep in industrial societies is corrupted by a growing trend of technological disruption and innovation [9] and high time-budget demands of the job market [10]. Specifically, our Bayesian inference predicted sleep duration to be approximately 0.75 h (45 min) longer in industrial populations compared with non-industrial populations. Furthermore, sleep efficiency was substantially greater among industrial populations, with a mean difference of 14% between the two groups. Indeed, our findings suggest the opposite of the sleep restriction epidemic hypothesis: rather than industrial society sleepers deviating from an optimal sleep state supposedly characteristic of ancestral populations, our results indicate that sleep duration and quality have, in fact, benefited from factors associated with industrialized socio-ecological contexts [53].

Our analysis strongly supports the circadian mismatch hypothesis, suggesting that the key issue in economically developed, industrial economies is not sleep duration but primarily circadian disruption. This is highlighted by the fact that non-industrial status significantly influences the model, predicting a 7% higher CFI in non-industrial societies compared with industrial ones. These findings suggest that interventions aimed at enhancing circadian function could have profound public health benefits. Specifically, they underscore the importance of developing policies and practices that align work and social schedules with natural circadian rhythms, potentially improving sleep quality, and by extension, overall health and wellness globally. This approach could mitigate the adverse effects of circadian misalignment, offering a strategic pathway to bolster public health—and ultimately health and wellness—around the globe.

Furthermore, to our knowledge, these are the first-ever estimates of species-level sleep parameters—including the most diverse representation of human societies for which sleep data are available—and can help guide future comparative work. Our analyses showed that the global human average for sleep duration is descriptively 6.78 h, and when the full model used in our analysis is considered, the predicted human sleep duration is 6.78 h with an 89% credible interval of 6.50−7.06 h. This corroborates recent longitudinal work demonstrating that polygenic risk scores for brain structure, cognition and mental health are compromised with (either insufficient or excessive) significant deviations from a 7 h optimum sleep duration [54]. Despite the well documented disruptive effect of electricity and lighting on both developing [55–58] and industrial societies [9], we suggest the advent of more secure, environmentally regulated sleep sites has been a driving factor that explains why both sleep duration and efficiency have increased in industrial societies. Yet, these same populations appear to be sleeping longer and more efficiently at the cost of desynchronization (i.e. inconsistency and fragmentation) of circadian rhythms, ultimately reducing circadian function. The very factor that may be improving sleep (e.g. secure, regulated sleep sites) may also be masking access to circadian entrainment— such as sunlight during the day, blue-light-emitting screens at night and outdoor temperature fluctuation. This suggests the intriguing possibility that there is an underexplored mismatch between circadian synchronization (i.e. a robust rhythm) and improved sleep duration and fragmentation for individuals who dwell in and outside industrialized contexts.

While sleep patterns in small-scale societies, both past and present, may not reflect an ‘optimized’ form of sleep [53], the persistence of hunter–gatherer subsistence strategies since the emergence of the genus *Homo* at approximately 1.8 Ma [59] suggests potential evolutionary advantages—such as more time to acquire skills and strengthen social networks—associated with shorter sleep durations throughout human evolution [60]. This suggests that the evolutionary history of reduced sleep duration in such societies might involve non-adaptive or context-specific benefits rather than direct health optimization. Critically, an evolutionary medicine perspective promotes the idea that natural selection operates on reproductive success, not optimized health [61]. In considering human sleep, clinical medicine’s emphasis on health optimization and evolutionary anthropology’s focus on reproductive benefits of sleep phenotypes both hold validity within their respective contexts. This nuanced view acknowledges that while health-focused recommendations are grounded in contemporary clinical outcomes, evolutionary perspectives highlight adaptive functions of sleep over historical time frames.

Studies assessing circadian function have revealed that more dysregulated/dampened rest–activity rhythms are associated with or predict negative health outcomes, including increased fatigue [62,63], depressive symptoms [45,62,64], obesity [48], risk of incident dementia and mild cognitive impairment [51], and mortality [65]. Evolutionary mismatch is a source of reduced wellness in places all over the world. As noted by Lloyd *et al*. [33, p. 3]: ‘A substantial proportion of human misery is probably due to genetic and cultural mismatch with our current environments’. Multiple negative health outcomes may be the result of the shift to dwelling in industrial societies characterized by climate-controlled, artificially extended photoperiods. Fortunately, they are outcomes that can in application be corrected to improve human wellness. As noted by Lane and colleagues in a 2022 review [23, p. 16]: ‘The fields of circadian rhythms and sleep are poised to make significant advances in human biology and improve human health’. To this end, we make the following recommendations.

As our comprehension of the perturbing impact of light and temperature on circadian rhythms expands, a viable avenue to enhance the well-being of contemporary sleepers emerges in the form of behavioural interventions. In this pursuit, it is crucial to maintain the advantageous foundation of secure, stable and well regulated sleep environments characteristic of industrial societies. Specifically, these interventions are aimed at ameliorating circadian function and improving individual and societal-level ‘chronohygiene’. Chronohygiene involves adopting behaviours that synchronize with an individual’s circadian rhythm to promote optimal well-being and sleep patterns [66]. This concept is not new, yet these novel comparative data strongly support the previous work performed by clinicians and research communities alike that have focused on circadian function in their patients and subjects. For example, previously recommended behavioural interventions encompass strategies such as structured daily routines, optimized exposure to natural light, and mindful engagement with electronic devices during evening hours, which can potentially mitigate the adverse impacts of circadian disruption and contribute to the overall enhancement of sleep quality and well-being.

This study has several limitations. In the Bayesian approximation analysis of sleep duration, only three forager populations were part of the analysis. Unfortunately, these data are likely not to expand dramatically in the near future given the continued force of globalization and resultant marginalization of indigenous groups practising traditional subsistence strategies. For the assessment of circadian function—which requires several days of consecutive analysis per individual—overcoming the paucity of reports of individual-level NPCRA parameters is a major challenge. Although circadian rhythm measurement has been applied in community-based samples [45,51]—including those with a number of disorders, including cancer [63], dementia [67] and psychiatric conditions [68,69]—the need for publicly accessible datasets that include representative samples, across varying cultures and modes of production, is urgent. Future research should focus on reporting and publishing measures of circadian rhythms using a wide variety of analytical techniques. Special consideration should be given to actigraphy-derived measures of circadian function, given the advantages of wide-scale, longitudinal application in ambulatory subjects. The testing of the circadian mismatch hypothesis is an important future research direction for sleep researchers to investigate and replicate these findings with a greater number of populations across differing latitudes and longitudes worldwide. We acknowledge the importance of caution when making generalized claims across globally distributed populations, considering the existing limitations in ecological and economic diversity across these regions.

The inclusion of studies with varying sample sizes and contextual factors underscores the need for careful interpretation of our findings in the context of the inherent heterogeneity present within and between these populations. Our analysis encompasses a broad range of populations and ecologies, introducing a limitation in conducting a detailed seasonal analysis due to the diverse access to light- and temperature-buffering technologies across these groups. Additionally, the omission of seasonal and temperature data in some studies within our dataset restricts our ability to fully explore the impact of these factors on sleep patterns. We recognize the substantial variability in sleep duration and patterns among non-industrial societies, such as the Tsimane and San, which highlights the need for future research to delve deeper into these variations and their ecological and cultural underpinnings [17].

Our analysis highlights several important considerations for future research into global sleep patterns. Future studies should aim to collect more granular data on latitude and seasonal changes in light–dark cycles, particularly for populations living at higher latitudes, as these factors likely play a critical role in shaping sleep and circadian patterns. Furthermore, the inclusion of genetic or ancestry data would greatly enhance our understanding of inter-population differences in sleep biology. Ancestry plays a well established role in determining chronotype, sleep architecture, and other sleep-related traits, but the lack of available genetic information in our dataset prevented us from analysing these factors. Future research that incorporates genetic, environmental and behavioural data will offer a more comprehensive picture of human sleep across different subsistence patterns and geographical locations. Addressing these gaps will not only refine our understanding of sleep ecology but also contribute to more tailored public health interventions for improving sleep in both industrial and non-industrial populations.

In summary, sleep is paramount to nearly every facet of our health and well-being. Sleep determines our ability to think (cognition), feel (emotional regulation), socialize (prosocial interaction) and preserve our health (immune strength and cellular repair and maintenance) [70]. It follows that sleep is one of the most powerful predictors of performance and wellness over the life-course. Thus, understanding how a rapidly changing world—by way of globalization, migration, new labour demands, and innovation in technological and economic systems—is affecting our sleep and circadian function is a critical twenty-first-century challenge. To this end, an interdisciplinary approach has emerged from anthropologists and sleep scientists. This new effort to ‘take the sleep lab into the field’ investigates how sleep in more ‘natural’ environments (where artificial light and technology play a lesser role) and ‘artificial’ urban environments (where artificial light and technology are more prevalent) differ in the ways in which they influence multiple dimensions of human health. This work underscores how cross-cultural comparisons, combined with an evolutionary medicine perspective, can enhance sleep, strengthen circadian function, and ultimately promote overall health and well-being.

## Data Availability

All data in Study 1 and materials necessary to reproduce our sleep duration analyses are available online in the Open Science Framework (OSF) data repository: https://osf.io/jy8ch/. As for Study 2 and the CFI analysis, the MIDUS raw actigraphy data used to generate circadian function measures (RA, IV, IS) are not available in the public domain. Unpublished MIDUS data access is restricted to the authors named in the approved request, and thus are ineligible for inclusion in the public data repository. Unpublished MIDUS data (including the raw actigraphy files used in this analysis) can be requested by submitting a letter to the MIDUS PI [71]. Supplementary material is available online [72].

## References

[1] Roenneberg T. 2013 Chronobiology: the human sleep project. Nature **498**, 427–428. (10.1038/498427a) 23803826

[2] Van Cauter E, Knutson KL. 2008 Sleep and the epidemic of obesity in children and adults. Eur. J. Endocrinol. **159**, S59–S66. (10.1530/eje-08-0298) 18719052 PMC2755992

[3] Colten HR, Altevogt B (eds)), Institute of Medicine Committee on Sleep Medicine and Research. 2006 Sleep disorders and sleep deprivation: an unmet public health problem, pp. 47–54. Washington, DC: National Academies Press. 20669438

[4] Hillman DR, Murphy AS, Pezzullo L. 2006 The economic cost of sleep disorders. Sleep **29**, 299–305. (10.1093/sleep/29.3.299) 16553015

[5] Hafner M, Stepanek M, Taylor J, Troxel WM, van Stolk C. 2017 Why sleep matters—the economic costs of insufficient sleep. A cross-country comparative analysis. Rand Health Q. **6**, 11. (10.7249/RR1791) PMC5627640 28983434

[6] Stranges S, Tigbe W, Gómez-Olivé FX, Thorogood M, Kandala NB. 2012 Sleep problems: an emerging global epidemic? Findings from the INDEPTH WHO-SAGE study among more than 40,000 older adults from 8 countries across Africa and Asia. Sleep **35**, 1173–1181. (10.5665/sleep.2012) 22851813 PMC3397790

[7] Samson DR. 2021 The human sleep paradox: the unexpected sleeping habits of Homo sapiens. Annu. Rev. Anthropol. **50**, 259–274. (10.1146/annurev-anthro-010220-075523)

[8] Yetish G, McGregor R. 2019 Hunter-gatherer sleep and novel human sleep adaptations. In Handbook of behavioral neuroscience, pp. 317–331. Amsterdam, The Netherlands: Elsevier. (10.1016/b978-0-12-813743-7.00021-9)

[9] Chang AM, Aeschbach D, Duffy JF, Czeisler CA. 2015 Evening use of light-emitting eReaders negatively affects sleep, circadian timing, and next-morning alertness. Proc. Natl Acad. Sci. USA **112**, 1232–1237. (10.1073/pnas.1418490112) 25535358 PMC4313820

[10] Chatzitheochari S, Arber S. 2009 Lack of sleep, work and the long hours culture: evidence from the UK Time Use Survey. Work Employ. Soc. **23**, 30–48. (10.1177/0950017008099776)

[11] Samson DR. 2020 Taking the sleep lab to the field: biometric techniques for quantifying sleep and circadian rhythms in humans. Am. J. Hum. Biol. **33**, e23541. (10.1002/ajhb.23541) 33252177

[12] Lamote de Grignon Pérez J, Gershuny J, Foster R, De Vos M. 2018 Sleep differences in the UK between 1974 and 2015: insights from detailed time diaries. J. Sleep Res. **28**, e12753. (10.1111/jsr.12753) 30198095 PMC6378586

[13] de la Iglesia HO *et al*. 2016 Ancestral sleep. Curr. Biol. **26**, R271. (10.1016/j.cub.2016.01.071) 27046809

[14] von Schantz M, Taporoski TP, Horimoto ARVR, Duarte NE, Vallada H, Krieger JE, Pedrazzoli M, Negrão AB, Pereira AC. 2015 Distribution and heritability of diurnal preference (chronotype) in a rural Brazilian family-based cohort, the Baependi study. Scient. Rep. **5**, 9214. (10.1038/srep09214) PMC4363835 25782397

[15] Ruiz FS *et al*. 2020 Early chronotype with advanced activity rhythms and dim light melatonin onset in a rural population. J. Pineal Res. **69**, e12675. (10.1111/jpi.12675) 32598502 PMC7508839

[16] Beijamini F *et al*. 2016 Timing and quality of sleep in a rural Brazilian family-based cohort, the Baependi Heart Study. Scient. Rep. **6**, 39283. (10.1038/srep39283) PMC5180217 28008932

[17] Yetish G, Kaplan H, Gurven M, Wood B, Pontzer H, Manger PR, Wilson C, McGregor R, Siegel JM. 2015 Natural sleep and its seasonal variations in three pre-industrial societies. Curr. Biol. **25**, 2862–2868. (10.1016/j.cub.2015.09.046) 26480842 PMC4720388

[18] Samson DR, Crittenden AN, Mabulla IA, Mabulla AZP, Nunn CL. 2017 Hadza sleep biology: evidence for flexible sleep-wake patterns in hunter-gatherers. Am. J. Phys. Anthropol. **162**, 573–582. (10.1002/ajpa.23160) 28063234

[19] Kilius E, Samson DR, Lew-Levy. S, Sarma. MS, Ouamba YR, Miegakanda V, Patel UA, Gettler LT, Boyette AH. 2021 Gender differences in BaYaka forager sleep-wake patterns in forest and village contexts. Scient. Rep. **11**, 13658. (10.1038/s41598-021-92816-6) PMC8249621 34211008

[20] Gettler LT, Samson DR, Kilius E, Sarma MS, Miegakanda V, Lew-Levy S, Boyette AH. 2023 Hormone physiology and sleep dynamics among BaYaka foragers of the Congo Basin: gendered associations between nighttime activity, testosterone, and cortisol. Horm. Behav. **155**, 105422. (10.1016/j.yhbeh.2023.105422) 37683498

[21] Gettler LT, Samson DR, Kilius E, Sarma MS, Ouamba YR, Miegakanda V, Boyette AH, Lew-Levy S. 2022 Links between household and family social dynamics with sleep profiles among BaYaka foragers of the Congo Basin. Social Sci. Med. **311**, 115345. (10.1016/j.socscimed.2022.115345) 36179483

[22] Prall SP, Yetish G, Scelza BA, Siegel JM. 2018 The influence of age- and sex-specific labor demands on sleep in Namibian agropastoralists. Sleep Health **4**, 500–508. (10.1016/j.sleh.2018.09.012) 30442317 PMC8110192

[23] Lane JM, Qian J, Mignot E, Redline S, Scheer F, Saxena R. 2022 Genetics of circadian rhythms and sleep in human health and disease. Nat. Rev. Genet. **24**, 4–20. (10.1038/s41576-022-00519-z) 36028773 PMC10947799

[24] Sateia MJ. 2014 International classification of sleep disorders—third edition: highlights and modifications. Chest **146**, 1387–1394. (10.1378/chest.14-0970) 25367475

[25] Widiger TA, Costa PT. 2013 Personality disorders and the five-factor model of personality, 3rd edn. Washington, DC: American Psychological Association.

[26] Pelayo R, Dement WC. 2017 History of sleep physiology and medicine. In Principles and practice of sleep medicine (eds MH Kryger, T Roth, WC Dement), pp. 3–14. Maryland Heights, MO: Elsevier. (10.1016/B0-72-160797-7/50008-2)

[27] Roenneberg T, Merrow M. 2016 The circadian clock and human health. Curr. Biol. **26**, R432–R443. (10.1016/j.cub.2016.04.011) 27218855

[28] Haus EL, Smolensky MH. 2013 Shift work and cancer risk: potential mechanistic roles of circadian disruption, light at night, and sleep deprivation. Sleep Med. Rev. **17**, 273–284. (10.1016/j.smrv.2012.08.003) 23137527

[29] Grimaldi D, Carter JR, Van Cauter E, Leproult R. 2016 Adverse impact of sleep restriction and circadian misalignment on autonomic function in healthy young adults. Hypertension **68**, 243–250. (10.1161/HYPERTENSIONAHA.115.06847) 27271308 PMC4902172

[30] Leproult R, Holmbäck U, Van Cauter E. 2014 Circadian misalignment augments markers of insulin resistance and inflammation, independently of sleep loss. Diabetes **63**, 1860–1869. (10.2337/db13-1546) 24458353 PMC4030107

[31] Bathgate CJ, Edinger JD, Wyatt JK, Krystal AD. 2015 Objective but not subjective short sleep duration associated with increased risk for hypertension in individuals with insomnia. Sleep **39**, 1037–1045. (10.5665/sleep.5748) PMC4835301 26951399

[32] Miller M, Cappuccio F. 2007 Inflammation, sleep, obesity and cardiovascular disease. Curr. Vasc. Pharmacol. **5**, 93–102. (10.2174/157016107780368280) 17430213

[33] Lloyd E, Wilson DS, Sober E. 2011 Evolutionary mismatch and what to do about it: a basic tutorial. Wesley Chapel, FL: Evolution Institute.

[34] Refinetti R. 2006 Circadian physiology, 2nd edn. Boca Raton, FL: CRC Press.

[35] Smith MR, Eastman CI. 2012 Shift work: health, performance and safety problems, traditional countermeasures, and innovative management strategies to reduce circadian misalignment. Nat. Sci. Sleep **4**, 111–132. (10.2147/NSS.S10372) 23620685 PMC3630978

[36] Harrison EM, Schmied EA, Yablonsky AM, Glickman GL. 2021 Implementation of interventions designed to promote healthy sleep and circadian rhythms in shiftworkers. Chronobiol. Int. **38**, 467–479. (10.1080/07420528.2020.1845190) 33327802

[37] Olson JA, Artenie DZ, Cyr M, Raz A, Lee V. 2020 Developing a light-based intervention to reduce fatigue and improve sleep in rapidly rotating shift workers. Chronobiol. Int. **37**, 573–591. (10.1080/07420528.2019.1698591) 31823658

[38] Samson DR, Manus MB, Krystal AD, Fakir E, Yu JJ, Nunn CL. 2017 Segmented sleep in a nonelectric, small-scale agricultural society in Madagascar. Am. J. Hum. Biol **29**, e22979. (10.1002/ajhb.22979) 28181718

[39] McKinnon L, Samson DR, Nunn CL, Rowlands A, Salvante KG, Nepomnaschy PA. 2022 Technological infrastructure, sleep, and rest-activity patterns in a Kaqchikel Maya community. PLoS One **17**, e0277416. (10.1371/journal.pone.0277416) 36383619 PMC9668134

[40] Patel UA, Gruen ME, Samson DR. 2021 A brief report of sleep and circadian rhythm quotas in a population of dog owners in North Carolina, USA. bioRxiv, 2021.01.21.427658. (10.1101/2021.01.21.427658)

[41] McElreath R. 2018 Statistical rethinking: a Bayesian course with examples in R and Stan. New York, NY: Chapman and Hall/CRC.

[42] R Core Team. 2023 R: a language and environment for statistical computing. Vienna, Austria: R Foundation for Statistical Computing. See https://www.R-project.org/.

[43] Bürkner PC. 2017 brms: An R package for Bayesian multilevel models using Stan. J. Stat. Softw. **80**, 1–28. (10.18637/jss.v080.i01)

[44] Carskadon MA, Dement WC. 2017 Normal human sleep: an overview. In Principles and practice of sleep medicine (eds MH Kryger, T Roth, WC Dement), pp. 15–24. Philadelphia, PA: Elsevier Saunders.

[45] Smagula SF, Ancoli-Israel S, Blackwell T, Boudreau R, Stefanick ML, Paudel ML, Stone KL, Cauley JA, Osteoporotic Fractures in Men (MrOS) Research Group. 2015 Circadian rest–activity rhythms predict future increases in depressive symptoms among community-dwelling older men. Am. J. Geriatr. Psychiatry **23**, 495–505. (10.1016/j.jagp.2014.06.007) 25066948 PMC4277502

[46] Ohayon M *et al*. 2017 National Sleep Foundation’s sleep quality recommendations: first report. Sleep Health **3**, 6–19. (10.1016/j.sleh.2016.11.006) 28346153

[47] Sivula T, Magnusson M, Vehtari A. 2022 Unbiased estimator for the variance of the leave-one-out cross-validation estimator for a Bayesian normal model with fixed variance. Commun. Stat. Theory Methods **52**, 5877–5899. (10.1080/03610926.2021.2021240)

[48] Luik AI, Zuurbier LA, Hofman A, Van Someren EJW, Tiemeier H. 2013 Stability and fragmentation of the activity rhythm across the sleep-wake cycle: the importance of age, lifestyle, and mental health. Chronobiol. Int. **30**, 1223–1230. (10.3109/07420528.2013.813528) 23971909

[49] McKinnon L, Samson DR, Nunn CL, Salvante KG, Nepomnaschy PA. 2022 Technological infrastructure, sleep, and rest-activity patterns in a Kaqchikel Maya community. Scient. Rep. **12**, 19694. (10.1038/s41598-022-24112-0) PMC9668134 36383619

[50] Radler BT. 2014 The Midlife in the United States (MIDUS) series: a national longitudinal study of health and well-being. Open Health Data **2**, e3. (10.5334/ohd.ai) 25558376 PMC4280664

[51] Tranah GJ *et al*. 2011 Circadian activity rhythms and risk of incident dementia and mild cognitive impairment in older women. Ann. Neurol. **70**, 722–732. (10.1002/ana.22468) 22162057 PMC3244839

[52] Ortiz-Tudela E, Martinez-Nicolas A, Campos M, Rol MÁ, Madrid JA. 2010 A new integrated variable based on thermometry, actimetry and body position (TAP) to evaluate circadian system status in humans. PLoS Comput. Biol. **6**, e1000996. (10.1371/journal.pcbi.1000996) 21085644 PMC2978699

[53] Nunn CN, Samson DR. 2019 Do we sleep better than our ancestors? San Antonio, FL: Evolution Institute.

[54] Li Y *et al*. 2022 The brain structure and genetic mechanisms underlying the nonlinear association between sleep duration, cognition and mental health. Nat. Aging **2**, 425–437. (10.1038/s43587-022-00210-2) 37118065

[55] de la Iglesia HO, Fernández-Duque E, Golombek DA, Lanza N, Duffy JF, Czeisler CA, Valeggia CR. 2015 Access to electric light is associated with shorter sleep duration in a traditionally hunter-gatherer community. J. Biol. Rhythm. **30**, 342–350. (10.1177/0748730415590702) PMC5320422 26092820

[56] Moreno CRC, Vasconcelos S, Marqueze EC, Lowden A, Middleton B, Fischer FM, Louzada FM, Skene DJ. 2015 Sleep patterns in Amazon rubber tappers with and without electric light at home. Scient. Rep. **5**, 14074. (10.1038/srep14074) PMC4566125 26361226

[57] Pilz LK, Levandovski R, Oliveira MAB, Hidalgo MP, Roenneberg T. 2018 Sleep and light exposure across different levels of urbanisation in Brazilian communities. Scient. Rep. **8**, 11389. (10.1038/s41598-018-29494-4) PMC6065379 30061685

[58] Smit AN, Broesch T, Siegel JM, Mistlberger RE. 2019 Sleep timing and duration in indigenous villages with and without electric lighting on Tanna Island, Vanuatu. Scient. Rep. **9**, 17278. (10.1038/s41598-019-53635-y) PMC6872597 31754265

[59] Dunsworth HM. 2010 Origin of the genus Homo. Evolution **3**, 353–366. (10.1007/s12052-010-0247-8)

[60] Samson DR, Nunn CL. 2015 Sleep intensity and the evolution of human cognition. Evol. Anthropol. **24**, 225–237. (10.1002/evan.21464) 26662946

[61] Stearns SC, Nesse RM, Govindaraju DR, Ellison PT. 2010 Evolutionary perspectives on health and medicine. Proc. Natl Acad. Sci. USA **107**, 1691–1695. (10.1073/pnas.0914475107) 20133821 PMC2868294

[62] Berger AM, Wielgus K, Hertzog M, Fischer P, Farr L. 2010 Patterns of circadian activity rhythms and their relationships with fatigue and anxiety/depression in women treated with breast cancer adjuvant chemotherapy. Support. Care Cancer **18**, 105–114. (10.1007/s00520-009-0636-0) 19381692

[63] Liu L *et al*. 2013 Fatigue and circadian activity rhythms in breast cancer patients before and after chemotherapy: a controlled study. Fatigue **1**, 12–26. (10.1080/21641846.2012.741782) 23412418 PMC3568994

[64] Raoux N, Benoit O, Dantchev N, Denise P, Franc B, Alliale JF, Widlöcher D. 1994 Circadian pattern of motor activity in major depressed patients undergoing antidepressant therapy: relationship between actigraphic measures and clinical course. Psychiatry Res. **52**, 85–98. (10.1016/0165-1781(94)90122-8) 8047624

[65] Tranah GJ *et al*. 2010 Circadian activity rhythms and mortality: the study of osteoporotic fractures. J. Am. Geriatr. Soc. **58**, 282–291. (10.1111/j.1532-5415.2009.02674.x) 20374404 PMC2938873

[66] Hildebrandt G. 1976 Outline of chronohygiene. Chronobiologia **3**, 113–127. 976021

[67] Hatfield CF. 2004 Disrupted daily activity/rest cycles in relation to daily cortisol rhythms of home-dwelling patients with early Alzheimer’s dementia. Brain **127**, 1061–1074. (10.1093/brain/awh129) 14998915

[68] Winkler D, Pjrek E, Praschak-Rieder N, Willeit M, Pezawas L, Konstantinidis A, Stastny J, Kasper S. 2005 Actigraphy in patients with seasonal affective disorder and healthy control subjects treated with light therapy. Biol. Psychiatry **58**, 331–336. (10.1016/j.biopsych.2005.01.031) 16102546

[69] Kasper S *et al*. 2010 Efficacy of the novel antidepressant agomelatine on the circadian rest-activity cycle and depressive and anxiety symptoms in patients with major depressive disorder: a randomized, double-blind comparison with sertraline. J. Clin. Psychiatry **71**, 109–120. (10.4088/JCP.09m05347blu) 20193645

[70] Walker MP. 2009 The role of sleep in cognition and emotion. Ann. NY Acad. Sci **1156**, 168–197. (10.1111/j.1749-6632.2009.04416.x) 19338508

[71] OSF HOME. 2022 Are humans undergoing a sleep epidemic or enlightenment? See https://osf.io/jy8ch/?view_only=9ea55e84e6e84ae4b56a811230128082.

[72] Samson DR, McKinnon L. 2025 Supplementary material from: Are humans facing a sleep epidemic or enlightenment? Large-scale, industrial societies exhibit long, efficient sleep yet weak circadian function. Figshare. (10.6084/m9.figshare.c.7669636)39999887

